# Supplementary material for: Chromosome-scale scaffolds of the fungus gnat genome reveal multi-Mb-scale chromosome-folding interactions, centromeric enrichments of retrotransposons, and candidate telomere sequences
Source: BMC Genomics. 2025 May 5;26:443. doi: 10.1186/s12864-025-11573-2 (PMC12051294; doi:10.1186/s12864-025-11573-2)
Supplement: Supplementary file 1 — Supplementary Material 1. [file 12864_2025_11573_MOESM1_ESM.pdf]

Supplemental Materials

Chromosome-scale scaffolds of the fungus gnat genome reveal multi-Mb-scale chromosome-folding interactions, centromeric enrichments of retrotransposons, and candidate telomere sequences.

John M. Urban[1], Susan A. Gerbi[2#], Allan C. Spradling[1#]

[1] Carnegie Institution for Science, Department of Embryology, Howard Hughes Medical Institute Research Laboratories, 3520 San Martin Drive, Baltimore, MD, 21218, USA

[2] Brown University Division of Biology and Medicine, Department of Molecular Biology, Cell Biology and Biochemistry, Providence, RI 02912 USA

[#] Co-senior authors contributed equally to this work.

Supplemental Figures

Supplemental Figure S1.

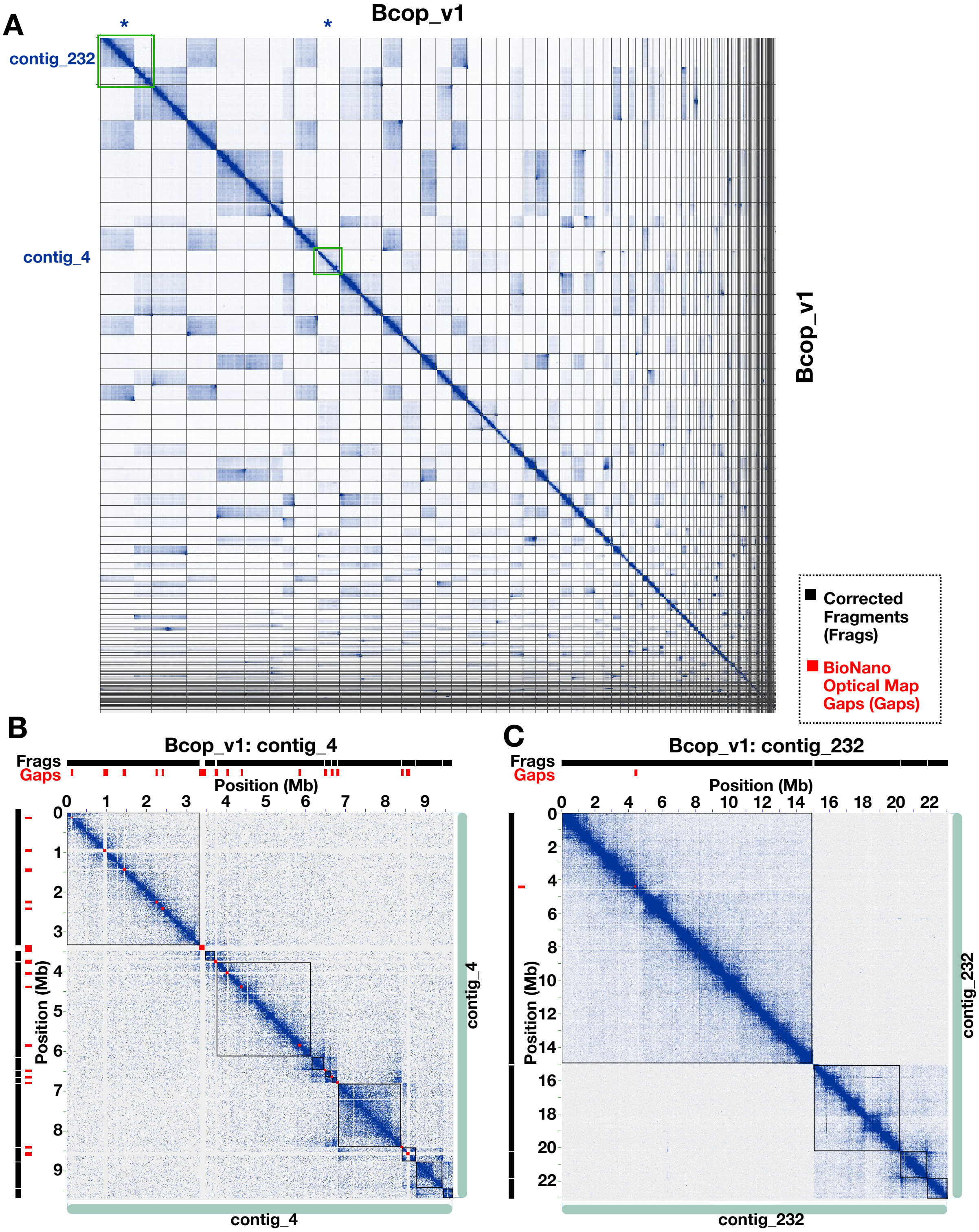

Supplemental Figure S1. Hi-C guided correction of contigs from Bcop_v1.

The paired-end Hi-C reads were first used to produce a corrected assembly (called "Bcop_v1_corrected") by aligning them to the 205 primary contigs of Bcop_v1 ("Bcop_v1_primary") [1], then manually inspecting the Hi-C interaction frequency signal within the Bcop_v1 contigs for disruptions in the expected pattern corresponding to putative mis-joined regions (**A-C**). **(A)** The entire Hi-C map of all primary scaffolds and contigs in Bcop_v1 ordered from longest to shortest before correction and scaffolding. Scaffolds from B and C are highlighted by green boxes. Bcop_v1 had 22 contigs with 46 such disruptions in the Hi-C signal (examples given in **B** and **C**, described below). The 22 contigs were "corrected" by breaking them into "sub-contigs" at the putative mis-joined regions. Of the 46 "debris" regions where breaks were made in Bcop_v1 contigs, 32 were adjacent to BioNano optical map gaps (example in **B**), i.e. regions joined together previously by BioNano optical maps in Bcop_v1, with gap lengths ranging from ~4.4 to ~674 kb. However, of the 156 BioNano gaps in Bcop_v1, there were 124 ranging from 25 bp to ~400 kb that were conserved after the correction step, 70 of which were on the same 22 contigs that had "debris" regions and 54 that were elsewhere within Bcop_v1. Thus, the regions joined by BioNano optical maps mostly did not present as putative mis-joins. Lower mapping quality from flanking repeats around BioNano gaps may have simply given rise to spurious Hi-C signals more often than random. In summary, the majority of mis-join signals (~70%) flanked a minority of assembly gaps, and mis-join signals affected a minority of contigs (10.7%) that were broken up by Hi-C-guided correction. This step transformed the input assembly (Bcop_v1_primary) that had 205 contigs into the corrected assembly (Bcop_v1_corrected), which had 297.

(B) Shows example of disrupted Hi-C signals related to joins made by scaffolding with BioNano Genomics optical maps and associated gaps. This is contig_4, a ~9.7 Mb contig with 14 optical map gaps that was broken into 10 corrected fragments with the 9 breaks correlated with gap

locations. New corrected contigs are outlined by black squares and optical map gaps are represented by red squares. Both are also displayed above and to the left of the Hi-C map. The bottom and right show seafoam-colored bars representing the full original Bcop_v1 contig.

(C) Example of disrupted Hi-C signal that occurred independent of optical map gaps within contiguous sequence. The Hi-C signal forms two obvious sequences that interact within themselves but that are significantly depleted of interactions with each other. The black squares outline the new contigs after correction. The self-interacting sequence on the bottom right has additional Hi-C signals that suggested possible structural errors; hence it being broken up into additional fragments. Fragments, optical map gaps, and the original contig are outlined and represented as in B.

Supplemental Figure S2.

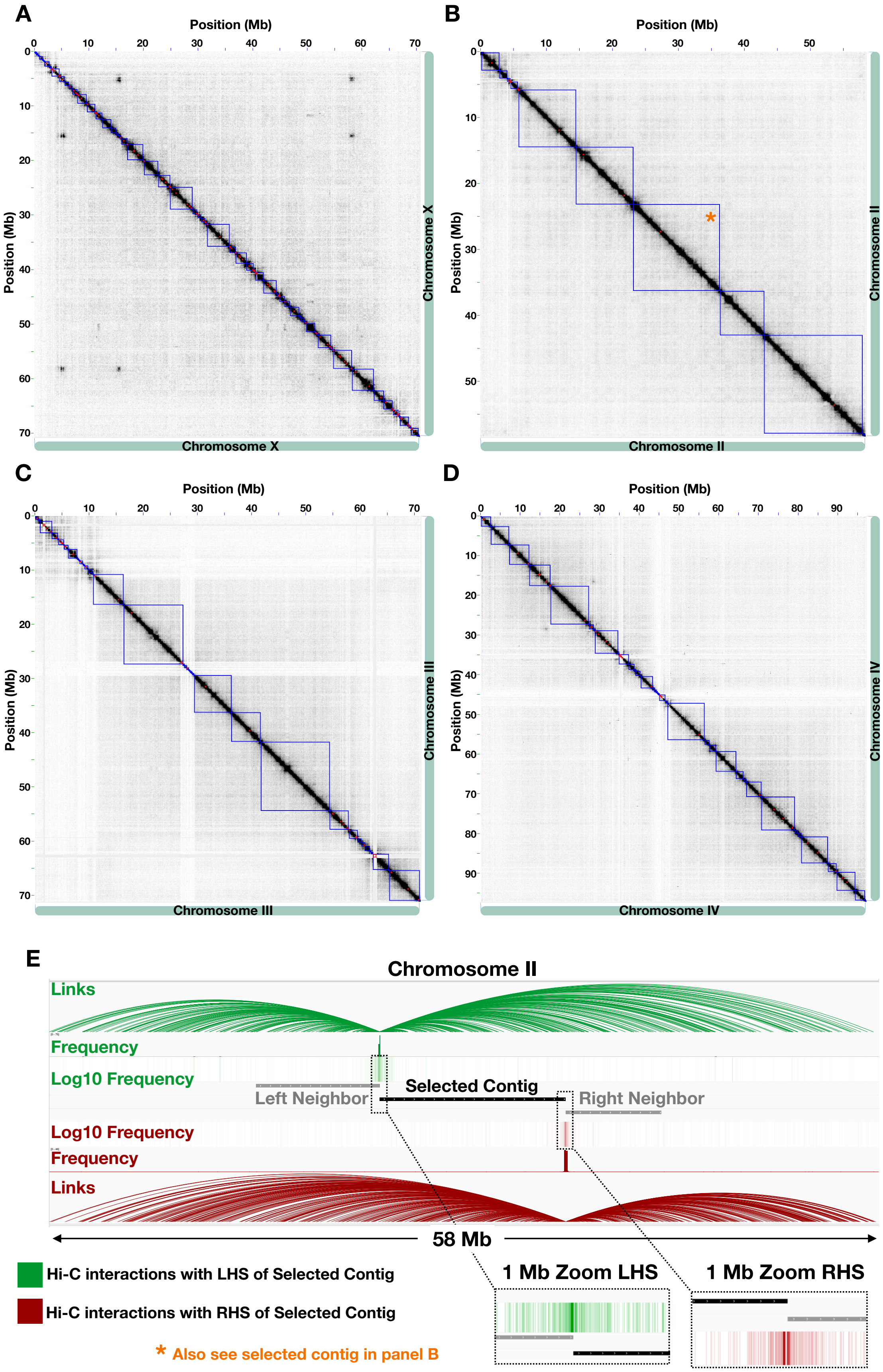

Supplemental Figure S2. Hi-C signal shows high 'chromosome specificity' and 'structural accuracy' of the chromosome-scale scaffolds.

The Hi-C signal across Bcop_v2 was manually inspected to ensure that these metrics were optimized according to expected interaction frequency patterns. First, intra-chromosomal interaction frequencies are expected to exceed inter-chromosomal interaction frequencies [2]. As such, for high 'chromosome specificity', the same should be true for interaction frequencies within scaffolds compared to between scaffolds. Second, within a chromosome, interaction frequencies are expected to be highest between adjacent loci and to decay with distance along the linear chromosome sequence [2]. As such, for high 'structural accuracy' within a given scaffold, short distances should have the highest interaction frequencies both within contigs and also between adjacent ends of neighboring contigs within the scaffold. The former was ensured in the previous pre-scaffolding "correction" step and the latter will hold true if the contigs were ordered and oriented correctly. In Figure 2A of the main paper, we showed that before scaffolding, the regions of high and low interaction frequencies are all over the map for the input into scaffolding (Bcop_v1_corrected) where contigs are simply ordered from longest to shortest. Then in Figure 2B of the main paper, we showed that after Hi-C scaffolding, the contigs are ordered such that those regions of high average interaction frequencies are confined within four chromosome-sized scaffold squares along the diagonal and the regions of lowest interaction frequency correspond to inter-scaffold contacts. This indicates that the contigs within a given scaffold correspond to the same chromosome and that different scaffolds correspond to different chromosomes. Here we zoom in on the individual chromosome-scale scaffolds to inspect the contigs and contig ordering that defines them.

(A, B, C, D) Interaction frequencies across the X, II, III, and IV chromosome scaffolds, respectively. Blue squares correspond to individual contigs from Bcop_v1_corrected that make up the scaffolds. The asterisk in D corresponds to the selected contig in G. High structural accuracy

of Bcop_v2 is indicated by the strong Hi-C interaction frequency signal along the diagonal within the chromosome scaffolds, with no disruptions between neighboring contigs, and by the weaker off-diagonal interaction frequencies that rapidly decay with distance.

(E) An example of the interaction frequencies between the ends of a selected contig and the rest of the chromosome-scale scaffold. The selected contig shown was from chromosome II (see asterisk in panel B). The selected contig is colored black whereas the two neighboring contigs are grey. Links (arcs) tracks correspond to a summary of loci that were linked together by paired-end reads where at least one mate maps to the given contig end. The "Frequency" tracks are bar plots corresponding to the number of times each bin across the chromosome was linked to the given contig end. However, interaction frequency decays exponentially with distance. Thus, the "Log10 Frequency" across the chromosome is also shown as a heatmap, and zoom-ins of the Log10 Frequency heatmaps in 1 Mb regions centered on each contig end are shown for higher resolution. Green and red tracks correspond to interactions and interaction frequencies with the left end and the right end of the selected contig, respectively. In general, several contigs were selected for the analysis highlighted in **E**. For the start (or end) of each contig inspected, compared to than any other region of any other contig within the scaffold, there were orders of magnitude more interactions with (i) the immediately adjacent terminal of the nearest neighboring contig, and (ii) the immediately adjacent flanking region within the same contig (example in **E**). Overall, these results indicate that the adjacent contigs within a given scaffold correspond to adjacent loci in the chromosome according to the logic of Hi-C. LHS and RHS stand for left-hand side and right-hand side, respectively.

Supplemental Figure S3.

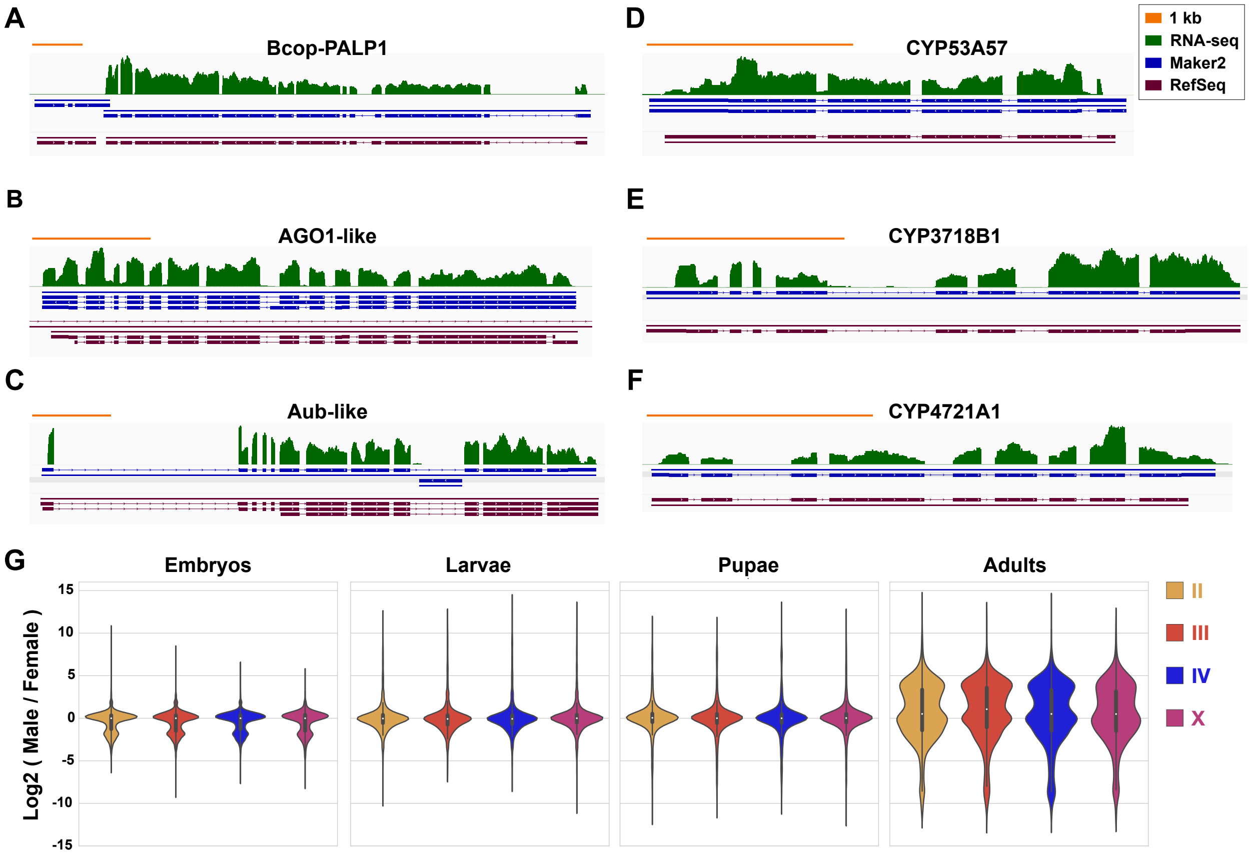

Supplemental Figure S3: Analysis of gene models on Bcop_v2 lifted over from Bcop_v1 gene annotation sets.

Two high quality gene annotation sets defined on the earlier version of the reference genome, Bcop_v1, were lifted over to the updated chromosome-scale reference, Bcop_v2. Both gene annotation sets had similar lift-over success rates for all metrics (Table 4). Using the Maker2 annotation set as an example, of the 23,117 genes and 28,870 transcripts, only 2 genes with 4 transcripts were not mapped to Bcop_v2. Of the 28,866 transcript models successfully lifted over to Bcop_v2, all but one (28,865) had perfect full-length alignments to the original transcript sequences, although 52 (<0.2%) were a bit shorter than the original. In total, only 53 transcript sequences had differences in the updated genome assembly (Table 4). Even fewer, just 49 transcripts, had differences at the level of protein sequence, which is to say that nearly all (99.83%) had identical protein sequences between assemblies (Table 4). An even higher percent at the gene level was identical between assemblies. For example, 99.87% of lifted-over genes had identical

protein sequences for all transcript isoforms, and 99.91% had identical protein sequences for all or at least a subset of transcript isoforms (Table 4). In addition to these analyses, we confirmed that RNA-seq coverage was concordant with the exons and introns of lifted over gene models.

(A-F) Example Bcop_v1 gene models were chosen to show concordance with RNA-seq datasets after liftover to Bcop_v2. For simplicity, only one RNA-seq dataset is shown (irradiated fourth instar pre-eyespot larvae, replicate 1 [3]). However, lifted over gene models were consistent with all RNA-seq datasets checked, including those from all life stages and both sexes used in the original gene annotation [1]. RNA-seq concordance is illustrated here using genes of interest from recent *B. coprophila* studies, including **(A-C)** PARP and Argonaute family genes that arose in response to radiation [3], and **(D-F)** example "alien" cytochrome P450 genes [4]. For all six gene panels **(A-F)**, as summarized in the pictorial legend box on the top right: the orange scale bar represents 1 kb, the green trace (top level) shows RNA-seq coverage, the blue gene models (middle level) are from Maker2 [1, 5], and the purple gene models (bottom level) are from NCBI RefSeq [1, 6]. In all cases, the RNA-seq trace shows genome-wide coverage that was computed agnostic to the location of gene structures, and that was not artificially restricted to exons. Thus, the correspondence of higher RNA-seq coverage over exons shows that fidelity of the liftover process. The gene model examples shown for RNA-seq coverage are as follows, adorned with Maker2 (Bcop_v1) [1, 5] and NCBI RefSeq (gene-LOC) [6] identifiers, either of which can be used with the lifted-over GFFs to find the coordinates in Bcop_v2. Bcop-PALP1: Bcop_v1_g007065; gene-LOC119085384. AGO1-like: Bcop_v1_g003309; gene-LOC119067568. Aub-like: Bcop_v1_g01302; gene-LOC119068172. CYP53A57: Bcop_v1_g020678; gene-LOC119075328. CYP3718B1: Bcop_v1_g007236; gene-LOC119074427. CYP4721A1: Bcop_v1_g009136; gene-LOC119078932.

(G) NCBI gene models lifted over to Bcop_v2 were used for chromosome-specific differential expression analysis of RNA-seq data from males versus females across four life stages [1]. Log2

fold-changes are shown for genes grouped by chromosome-scale scaffolds. The distribution of male vs. female fold-changes across X-linked genes match the distributions across each autosomal chromosome rather having a 2-fold down-shifted X-linked fold-change distribution corresponding to the single male X chromosome compared to two in females. This indicates there is dosage compensation on the single male X as we and others found previously [1, 7].

Supplemental Figure S4.

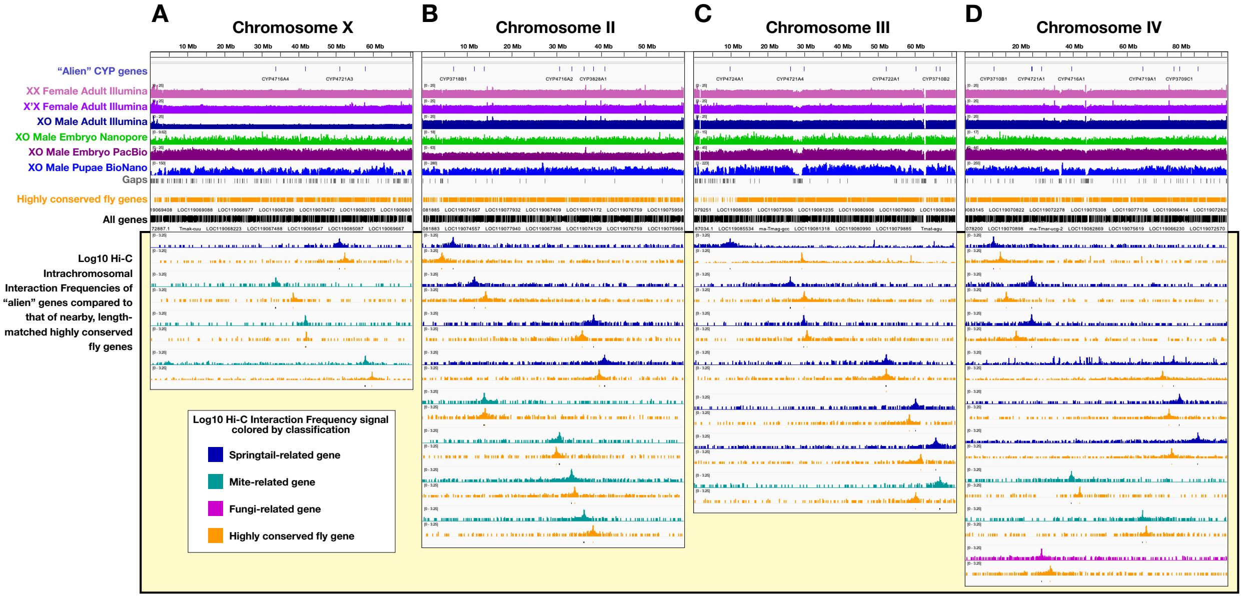

Supplemental Figure S4: Diverse genomic datasets support the existence of "alien" genes within the chromosomes.

A recent paper found 28 "alien" genes from horizontal gene transfer (HGT) in the Bcop_v1 gene annotations, 17 from springtails, 10 from mites, and 1 from fungi. They presented some evidence that these are not from contamination sources [4]. For example, the "alien" genes had homology and synteny in the genomes of closely related species from three different continents and had evidence of being expressed and spliced [4], which we also show for three such genes in Supplemental Fig. S3 D-F. The locations of the "alien" genes in the chromosome-scale scaffolds allowed a further investigation into the likeliness that these 'alien' genes are truly integrated into the chromosomes of *B. coprophila* with a variety of orthogonal genomic datasets, including Hi-C, long reads, and optical maps. As shown in **A-D** (i) they have typical Hi-C interaction frequencies that decay with distance, comparable in all cases to nearby 'highly conserved fly genes' of similar length, and (ii) there were no disruptions in sequencing depth seen over the "alien" gene regions across a variety of genomic datasets from different technologies and samples whereas disruptions were seen over gaps in the assembly as expected. Overall, the genes reported to be "aliens" by Feyersen et al [4] show no differences in coverage

or Hi-C interactions compared to highly conserved fly genes, demonstrating their existence inside the scaffolds is not a result of contamination and/or assembly errors.

IGV traces for chromosome **(A)** X, **(B)** II, **(C)** III, and **(D)** IV. From the top track downward, each shows the location of (1) “alien” CYP genes described by Feyersen et al [4], (2-7) coverage tracks from various genomic technologies and samples [1, 8], (8) gaps in the assembly where coverage signal is expected to drop, (9) locations of all highly conserved fly genes, and (10) locations of all genes. In each, the 11th track from the top and all tracks beneath it highlighted by the yellow box correspond to the log10 intrachromosomal interaction frequency (IIF) signal emanating from selected “alien” genes (dark blue, cyan, or magenta) or the closest by highly conserved fly gene that is approximately the same length (orange). All log10 IIF tracks have the same y-axis range (0-3.25), allowing direct comparison of peak heights and shapes. Under each pair of log10 IIF signals is a track showing the exact location of both genes.

Supplemental Figure S5.

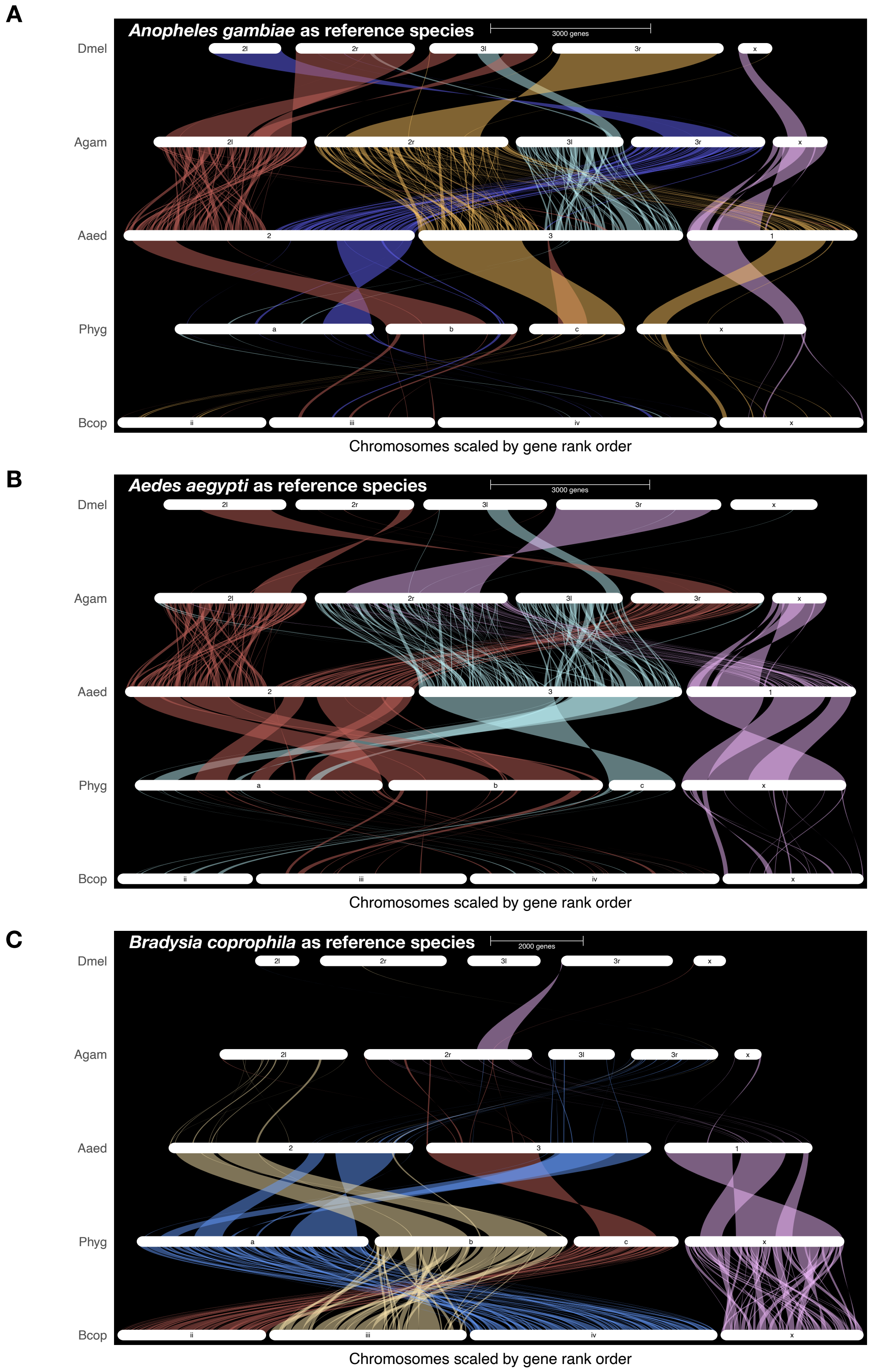

Supplemental Figure S5. Riparian plots of syntenic blocks.

(A) Riparian plot of syntenic block between the 5 species using *Anopheles gambiae* as the reference. **(B)** Riparian plot of syntenic block between the 5 species using *Aedes aegypti* as the reference. **(C)** Riparian plot of syntenic block between the 5 species using *Bradysia coprophila* as the reference. Using these three as references was sufficient to visually capture all the trends of syntenic blocks between species. Also see Fig. 3 and Table 5.

Supplemental Figure S6. Most of the extra long-range dots in X'X female data are from structural differences between X and X', and are not found in the XO male data.

A

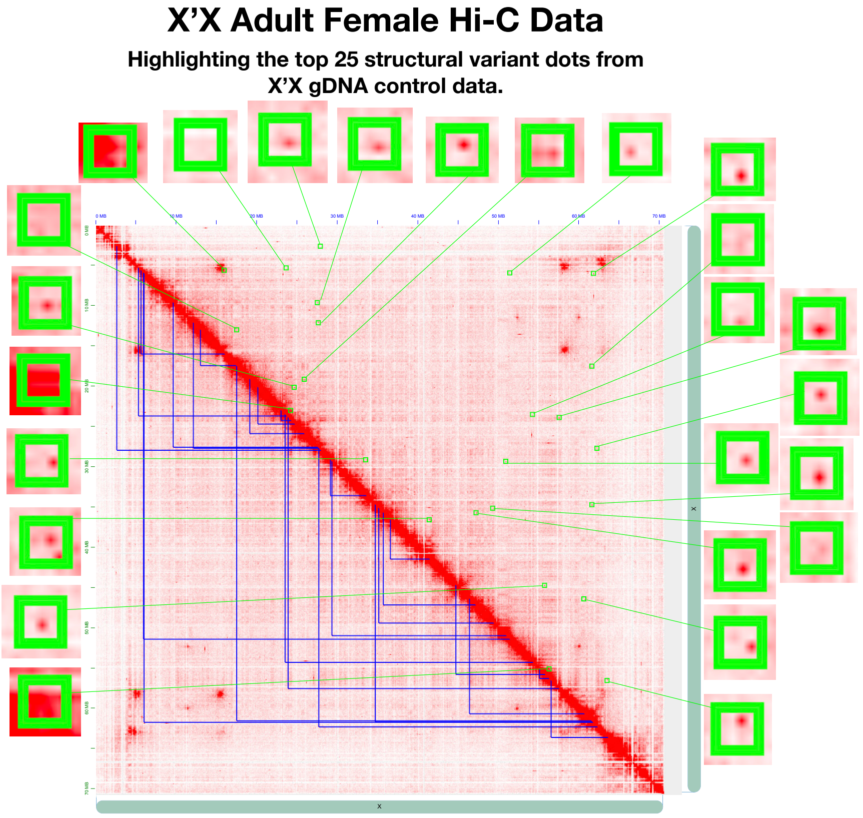

B

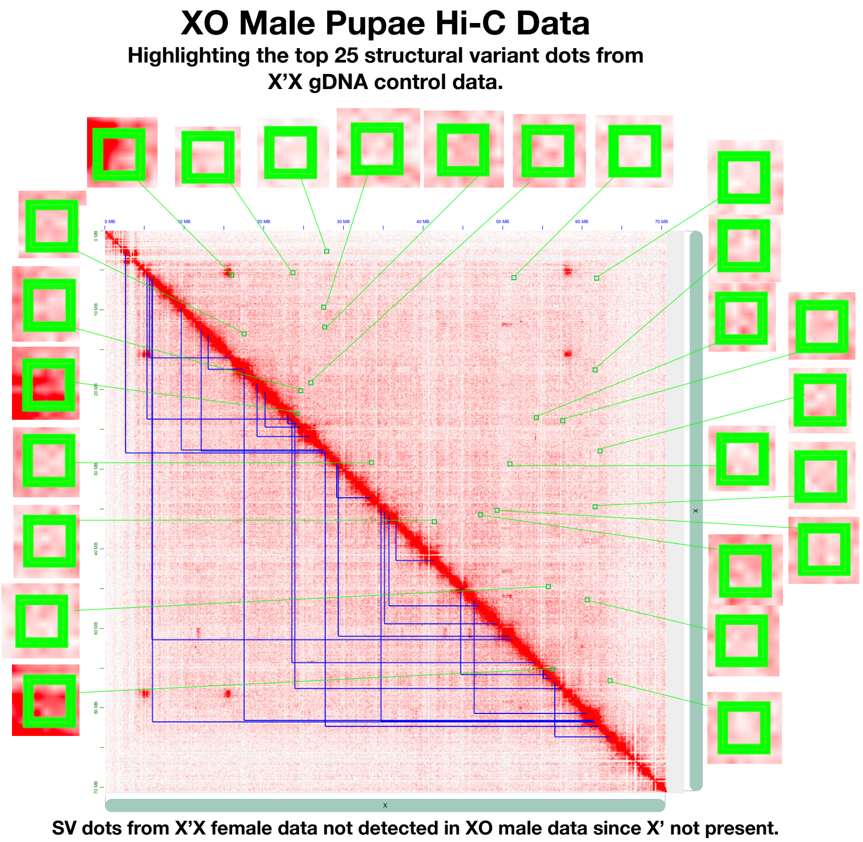

Supplemental Figure S6. Most of the extra long-range dots in X’X female data are from structural differences between X and X’, and are not found in the XO male data.
The X’X female Hi-C map has many additional off-diagonal dots than the XO male Hi-C map. We suspected this was due to structural differences between the X’ and X chromosomes. We used regular X’X genomic DNA (gDNA) paired-end sequencing data from a recent study [8] with a similar approach of calling long-range (> 5 Mb) interaction peaks as done for identifying fold-back regions and inversion breakpoints in the X’X Hi-C data. This identified many of the extra dots on the X’X Hi-C map thought to be putative structural difference between the X’ and X chromosomes. Here, the top 25 cluster peaks of gDNA SVs were used to annotate X’X and XO Hi-C plots to check how many off-diagonal dots are seen in each Hi-C map **(A)** X’X Adult Female Hi-C map with the 25 gDNA SV locations highlighted by green squares, which are also blown up around the perimeter of the Hi-C map. The majority of green squares (SV locations) have obvious visible dots inside them. A couple green squares do not, possibly due to smoothing or differences between the X’X gDNA and X’X Hi-C samples. **(B)** XO Male Pupae Hi-C map with the 25 gDNA SV locations highlighted by green squares as in (A). The X’X dots are not visible in XO male Hi-C data. The blue half-squares on the bottom-left half of the map show how the loci are connected, and the dots are covered by the corners. The green squares in the top-right half of the map are drawn around the dots of interest.

Supplemental Figure S7. Examples of 7 other putative long-range interactions on the X.

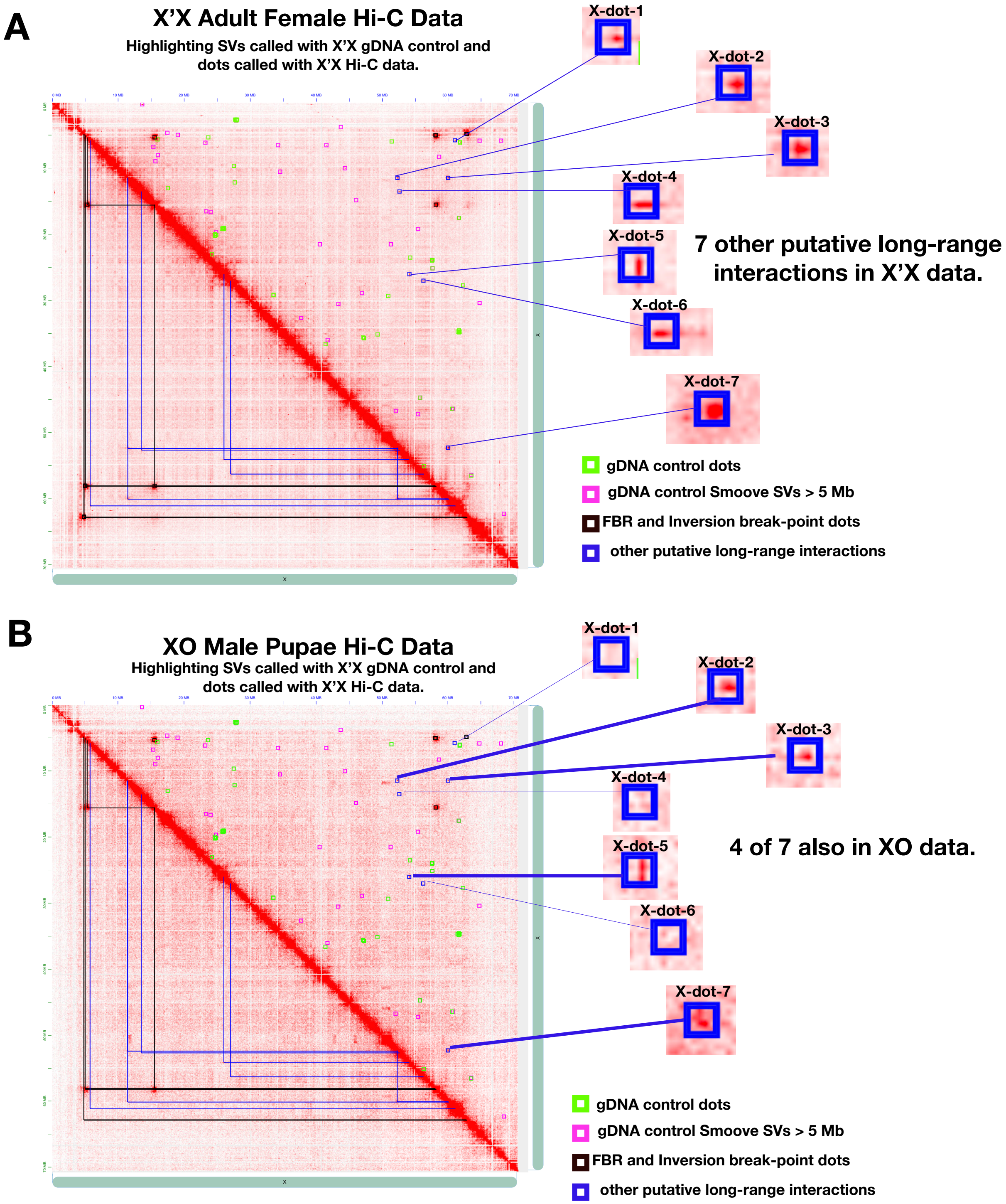

Supplemental Figure S7. Examples of 7 other putative long-range interactions on the X. We searched for long-range interactions other than the strong, broad interactions demonstrated by the fold-back regions and long paracentric inversion breakpoints. First, many cluster peaks were called on the X'X Hi-C map. In addition, X'X genomic DNA control data was used to call structural variant cluster peaks similar to Supplemental Figure S7, but allowing up to 50 calls. In addition, structural variants in the X'X gDNA control data were called with Smoove/Lumpy [9, 10]. Then X'X Hi-C interactions were filtered to remove any possible SV as well as the FBR and long paracentric inversion regions. There were 7 dots that remained, 4 of which are also in the XO male data. **(A)** X'X Adult Female Hi-C map with FBR, long paracentric inversion breakpoint, SV locations, and extra putative long-range interactions highlighted. **(B)** XO Male Pupae Hi-C map highlighting the locations of FBR, long paracentric inversion breakpoint, SV locations, and extra putative long-range interactions, all of which were learned on the X'X datasets. The seven putative long-range interaction dots on the X are simply named X-dot 1-7. X-dot-2, X-dot-3, X-dot-5, and X-dot-7 were also seen in the male pupae Hi-C data. The blue half-squares on the bottom-left half of the map show how the extra dot loci are connected, and the black squares do the same for the fold-back regions and breakpoints for the long paracentric inversion. The squares in the top-right half of the map are drawn around the dots of interest, and colored by what they represent as shown in the legends. The blue squares are blown up to show the extra dot locations in both Hi-C datasets.

Supplemental Tables

Supplemental Table S1. Genes within broadly-defined FBR1 boundaries.

Chr	Start	End	ID	Associated Functional Information
X	4635542	4692595	LOC119069868	neurexin-3a-like
X	4732320	4793358	LOC119067090	uncharacterized LOC119067090
X	4798989	4802436	LOC119067096	lncRNA
X	4808800	4837292	LOC119067083	uncharacterized LOC119067083
X	4840265	4841893	LOC119067085	beclin-1-like protein
X	4847876	4854355	LOC119067074	probable histone-lysine N-methyltransferase Mes-4
X	4862162	4865889	LOC119067081	FAST kinase domain-containing protein 4
X	4865845	4869812	LOC119067079	IQ and ubiquitin-like domain-containing protein
X	4881319	4894576	LOC119067082	protein phosphatase PP2A 55 kDa regulatory subunit
X	4896492	4899359	LOC119067077	TATA box-binding protein-associated factor RNA polymerase I subunit B
X	4903023	4919416	LOC119067080	inositol polyphosphate-5-phosphatase A
X	4920112	4922755	LOC119067078	extracellular xylan exo-alpha-(1->2)-glucuronosidase-like
X	4933633	4936136	LOC119067091	max-like protein X
X	4936065	4937668	LOC119067087	tRNA:m(4)X modification enzyme TRM13 homolog
X	4939081	4940231	LOC119067093	transmembrane emp24 domain-containing protein 2
X	4948412	4951566	LOC119067089	5-hydroxytryptamine receptor 1-like
X	4955521	4996863	LOC119067088	5-hydroxytryptamine receptor 1-like
X	5016878	5076694	LOC119067075	mushroom body large-type Kenyon cell-specific protein 1
X	5217451	5232532	LOC119067094	lncRNA
X	5261380	5279772	LOC119067095	lncRNA
X	5391430	5397915	LOC119066998	uncharacterized LOC119066998
X	5440820	5451531	LOC119066997	uncharacterized LOC119066997
X	5456715	5463343	LOC119067026	ras-related protein Rab-1A
X	5470204	5475173	LOC119067031	chondroitin sulfate glucuronyltransferase
X	5476547	5490089	LOC119067027	ATP-binding cassette sub-family A member 3-like
X	5493693	5495585	LOC119066995	exosome complex component RRP45-like
X	5508837	5519504	LOC119067025	plastin-1-like
X	5522843	5528958	LOC119067010	ER membrane protein complex subunit 1
X	5530688	5532706	LOC119067009	protein farnesyltransferase subunit beta
X	5533800	5545432	LOC119067008	cyclin G
X	5550449	5550761	LOC119067007	lncRNA
X	5557620	5561141	LOC119067005	uncharacterized LOC119067005
X	5564541	5570403	LOC119066994	kinesin-like protein Nod
X	5571313	5574392	LOC119067014	uncharacterized LOC119067014
X	5572958	5573449	LOC119067015	lncRNA
X	5583226	5588388	LOC119067017	uncharacterized LOC119067017
X	5598102	5639348	LOC119067016	regulator of G-protein signaling 9
X	5645805	5665544	LOC119066993	uncharacterized LOC119066993
X	5690809	5697035	LOC119070114	cytochrome c1
X	5699343	5700120	LOC119070109	lncRNA
X	5707253	5722892	LOC119070126	plastin-1
X	5726821	5729416	LOC119070142	histone deacetylase complex subunit SAP30 homolog
X	5731320	5738467	LOC119070124	protein UBASH3A homolog
X	5739860	5744992	LOC119070144	uncharacterized LOC119070144

X	5750829	5754225	LOC119070135	nuclear transcription factor Y subunit gamma-like
X	5825210	5838009	LOC119070150	lncRNA
X	5831123	5860429	LOC119070117	ras-responsive element-binding protein 1
X	5842684	5844659	LOC119070129	protein I'm not dead yet-like

FBR1 was defined as X:4617817-5858425:FBR1. The FBR regions used were as broadly defined as possible to include all possible genes of interest. The coordinates of each gene within the region is given. Gene ID corresponds to NCBI's RefSeq annotation of Bcop_v1, but the gene coordinates are shown for Bcop_v2. The associated functional information is simply what putative orthologous protein assignment was given by NBCI during their annotation process.

Supplemental Table S2. Genes within broadly-defined FBR2 boundaries.

Chr	Start	End	ID	Associated Functional Information
X	14941994	14980081	LOC119070023	mucin-19
X	15020528	15025840	LOC119070028	telomerase-binding protein EST1A
X	15025879	15026846	LOC119070029	ribosome maturation protein SBDS
X	15028467	15035685	LOC119070026	protein phosphatase Slingshot
X	15029173	15029731	LOC119070027	lncRNA
X	15044391	15052048	LOC119070061	glutamate receptor ionotropic kainate 2
X	15058881	15072732	LOC119070063	uncharacterized LOC119070063
X	15085802	15088529	LOC119070064	uncharacterized LOC119070064
X	15090150	15092181	LOC119070041	uncharacterized LOC119070041
X	15093008	15095408	LOC119070055	uncharacterized LOC119070055
X	15098283	15099010	LOC119070056	lncRNA
X	15105248	15110168	LOC119070054	alpha/beta hydrolase domain-containing protein 17B-like
X	15112056	15114506	LOC119070036	solute carrier family 25 member 45-like
X	15117031	15118828	LOC119070038	mitochondrial basic amino acids transporter-like
X	15120198	15121710	LOC119070060	2-methylene-furan-3-one reductase-like
X	15122667	15124189	LOC119070059	uncharacterized LOC119070059
X	15125094	15131673	LOC119070057	protein GPR107
X	15139899	15302569	LOC119070042	uncharacterized LOC119070042
X	15149555	15158633	LOC119070050	lncRNA
X	15172267	15173730	LOC119070045	poly(3-hydroxyalkanoate) depolymerase C-like
X	15187588	15197624	LOC119070049	parkin coregulated gene protein homolog
X	15208761	15210819	LOC119070043	uncharacterized LOC119070043
X	15300963	15313890	LOC119070046	mitochondrial dicarboxylate carrier
X	15303880	15310855	LOC119070047	uncharacterized LOC119070047
X	15341498	15435604	LOC119070065	pyrokinin-1 receptor-like
X	15436104	15438709	LOC119070066	T-complex protein 1 subunit alpha-like
X	15451951	15466295	LOC119070052	glutamyl aminopeptidase
X	15521731	15523213	LOC119069401	uncharacterized LOC119069401
X	15528862	15529735	LOC119069400	uncharacterized LOC119069400
X	15532893	15534365	LOC119069398	upstream activation factor subunit spp27
X	15626340	15628004	LOC119070108	uncharacterized LOC119070108
X	15650107	15650325	LOC119070107	lncRNA
X	15651083	15651301	LOC119070106	lncRNA
X	15672405	15678494	LOC119070102	sodium-independent sulfate anion transporter-like
X	15678827	15681019	LOC119070103	uncharacterized LOC119070103
X	15683470	15684974	LOC119070105	trypsin alpha-3-like
X	15685777	15688809	LOC119070104	trypsin-like
X	15691863	15692322	LOC119070040	uncharacterized N-acetyltransferase YjgM-like
X	15696063	15699160	LOC119070101	STAM-binding protein
X	15705364	15706167	LOC119070099	lncRNA
X	15709070	15721953	LOC119070098	calcium-binding mitochondrial carrier protein Aralar1
X	15723383	15724930	LOC119070097	uncharacterized LOC119070097
X	15729684	15750334	LOC119070096	corticotropin-releasing factor-binding protein
X	15737294	15741918	LOC119070095	uncharacterized LOC119070095
X	15752952	15758923	LOC119070094	probable peroxisomal acyl-coenzyme A oxidase 1
X	15773943	15798358	LOC119070093	lncRNA
X	15775352	15797304	LOC119070091	acyl-CoA desaturase-like
X	15800630	15802787	LOC119070092	venom serine protease-like
X	15808932	15809300	LOC119070090	lncRNA

X	15809429	15810572	LOC119070088	DNA polymerase interacting tetratricopeptide repeat-containing protein of 47 kDa
X	15811078	15812750	LOC119070087	probable phenylalanine--tRNA ligase mitochondrial
X	15814469	15816269	LOC119070086	trans-Golgi network integral membrane protein 2-like
X	15830359	15832130	LOC119070039	tRNA pseudouridine(38/39) synthase;start_range=.,176999
X	15835426	15835776	LOC119070089	lncRNA
X	15839985	15853681	LOC119070084	ubiquitin carboxyl-terminal hydrolase puf
X	15856802	15858611	LOC119070083	uncharacterized LOC119070083
X	15859818	15862488	LOC119070082	presenilin homolog
X	15863724	15866154	LOC119070081	WD40 repeat-containing protein SMU1
X	15867872	15872533	LOC119070080	alpha-mannosidase 2
X	15876532	15944404	LOC119070077	espin
X	15886314	15887402	LOC119070079	uncharacterized protein C9orf85 homolog
X	15946809	15949511	LOC119070071	another transcription unit protein
X	15949503	15950687	LOC119070074	tRNA (cytosine(38)-C(5))-methyltransferase
X	15951281	15956954	LOC119070073	uncharacterized LOC119070073
X	15964775	15970332	LOC119070076	probable nuclear transport factor 2
X	15971308	15972414	LOC119070075	haloacid dehalogenase-like hydrolase domain-containing protein 2
X	15972501	15978458	LOC119070069	splicing factor 3B subunit 1
X	15981523	15985621	LOC119070072	uncharacterized LOC119070072
X	15987288	15992222	LOC119070068	actin-5C
X	16016851	16020658	LOC119068117	syndetin
X	16027366	16029709	LOC119068062	lncRNA
X	16056213	16113642	LOC119068048	uncharacterized LOC119068048

FBR2 was defined as X:14956213-16058975:FBR2. The FBR regions used were as broadly defined as possible to include all possible genes of interest. The coordinates of each gene within the region is given. Gene ID corresponds to NCBI's RefSeq annotation of Bcop_v1, but the gene coordinates are shown for Bcop_v2. The associated functional information is simply what putative orthologous protein assignment was given by NBCI during their annotation process.

Supplemental Table S3. Genes within broadly-defined FBR3 boundaries.

Chr	Start	End	ID	Associated Functional Information
X	57743177	57759852	LOC119085695	probable cytochrome P450 28d1
X	57754722	57777203	LOC119085691	sodium-coupled monocarboxylate transporter 1-like
X	57762921	57767083	LOC119085693	sodium-coupled monocarboxylate transporter 1-like
X	57768291	57779281	LOC119085698	lncRNA
X	57772527	57774533	LOC119085697	sodium-coupled monocarboxylate transporter 1-like
X	57786916	57788756	LOC119085655	serine/threonine-protein kinase Warts-like
X	57796957	57797892	LOC119085654	serine/threonine-protein kinase Warts-like
X	57804904	57858764	LOC119085644	serine/threonine-protein kinase Warts-like
X	57817368	57830424	LOC119085645	lncRNA
X	57861375	57861864	LOC119080333	ctenidin-1-like
X	57863966	57865369	LOC119085728	WD repeat-containing protein 61
X	57865427	57866518	LOC119085729	protein NDUFAF4 homolog
X	57869843	57870527	LOC119085721	uncharacterized LOC119085721
X	57872522	57873422	LOC119085653	uncharacterized LOC119085653
X	57874301	57882770	LOC119085706	neuralized-like protein 4
X	57879098	57879170		tRNA
X	57897190	57897262		tRNA
X	57917771	57918430	LOC119085643	lncRNA
X	57932507	57938134	LOC119085713	cytochrome P450 3A2-like
X	57938378	57949865	LOC119085711	cytochrome P450 4d1-like
X	57951304	57972365	LOC119085714	lncRNA
X	57951445	57954026	LOC119085712	cytochrome P450 4d1-like
X	58014747	58015823	LOC119080397	thiopurine S-methyltransferase-like
X	58077825	58102369	LOC119081066	lncRNA
X	58091753	58092418	LOC119081059	lncRNA
X	58156161	58159064	LOC119085652	uncharacterized LOC119085652
X	58188826	58243636	LOC119081069	F-actin-monooxygenase Mical
X	58195622	58202717	LOC119081098	carboxymethylenebutenolidase homolog
X	58245423	58256907	LOC119081088	palmitoyltransferase ZDHHC6
X	58264482	58289540	LOC119080403	acetylcholinesterase-like;start_range=.,2701
X	58293917	58322925	LOC119069722	acetylcholinesterase
X	58348605	58363237	LOC119069708	CRISP/Allergen/PR-1
X	58366187	58607377	LOC119069857	protein timeless homolog
X	58440299	58443346	LOC119069858	calcium-activated chloride channel regulator 1-like
X	58483092	58506981	LOC119069862	lncRNA
X	58485475	58527685	LOC119069856	uncharacterized LOC119069856
X	58530201	58530918	LOC119069861	lncRNA
X	58612542	58614675	LOC119069859	endoglucanase A-like
X	58646655	58647918	LOC119069860	uncharacterized LOC119069860
X	58648914	58649948	LOC119069855	leucine-rich repeat-containing G-protein coupled receptor 5-like
X	58650746	58651781	LOC119069854	uncharacterized LOC119069854

FBR3 was defined as X:57757173-58653168:FBR3. The FBR regions used were as broadly defined as possible to include all possible genes of interest. The coordinates of each gene within the region is given. Gene ID corresponds to NCBI's RefSeq annotation of Bcop_v1, but the gene coordinates are shown for Bcop_v2. The associated functional information is simply what putative orthologous protein assignment was given by NBCI during their annotation process.

Supplemental Table S4. Narrow estimates of interacting regions using interaction frequency summits.

Region	Summit Coordinates	Summit Candidate	Support	Supporting Interactions
**5pINVbp**	**X:4856672-4891117**	**1**	**1/1**	**XpX-INV-1**
**FBR1**	**X:4994450-5028895**	**1**	**3/5**	**XpX-FBR1-FBR3-1, XO-FBRas1-FBR3-1, XO-FBR1b-FBR2-1**
FBR1	X:5270006-5304451	2	1/5	XpX-FBR1-FBR2-1
FBR1	X:5304451-5338895	3	1/5	XO-FBR1a-FBR2-1
**FBR2**	**X:15500019-15534463**	**1**	**3/5**	**XO-FBR2-FBR3-1, XpX-FBR1-FBR2-2, XpX-FBR2-FBR3-1**
FBR2	X:15534463-15568907	2	1/5	XO-FBR1b-FBR2-2
FBR2	X:15603352-15637796	3	1/5	XO-FBR1a-FBR2-2
**FBR3**	**X:58142292-58176736**	**1**	**2/4**	**XpX-FBR1-FBR3-2, XO-FBR1-FBR3-2**
FBR3	X:58176736-58211181	2	1/4	XO-FBR2-FBR3-2
FBR3	X:58211181-58245625	3	1/4	XpX-FBR2-FBR3-2
**3pINVbp**	**X:62861186-62895631**	**1**	**1/1**	**XpX-INV-2**

These are narrow estimates of the pairs of loci that interact. Within broader domains (cluster peaks), these are the sub-regions with the highest interaction frequencies (or peak summits). There were two Hi-C samples analyzed: XO male pupae (XO) and X'X adult females (XpX). There is a single summit estimate for each inversion breakpoint. However, each FBR is involved in two interactions per sample across two samples. Moreover, we took two estimates for FBR1 from the male XO sample since the second candidate matched that learned from the XpX sample. Thus, there could mean up to 4 summit candidates each FBR. The support number refers to the fraction of supporting interactions across samples that gave the same summit. The Supporting Interactions detail what samples and what interacting regions supported the summit. For each region, the summit with strongest support is shown in bold.

Supplemental Table S5. Genes at FBR and long paracentric inversion breakpoint interaction frequency summits.

Interaction Region	Summit Candidate	Gene Distance	Gene ID	Gene Coordinates	Associated Functional Info
5pINVbp	1	0	LOC119067079	X:4865845-4869812	IQ and ubiquitin-like domain-containing protein
5pINVbp	1	0	LOC119067081	X:4862162-4865889	FAST kinase domain-containing protein 4
5pINVbp	1	0	LOC119067082	X:4881319-4894576	protein phosphatase PP2A 55 kDa regulatory subunit
FBR1	1	0	LOC119067075	X:5016878-5076694	mushroom body large-type Kenyon cell-specific protein 1
FBR1	1	0	LOC119067088	X:4954286-4996863	5-hydroxytryptamine receptor 1-like
FBR1	2	0	LOC119067095*	X:5261380-5279772	lncRNA
FBR1	3	-24680	LOC119067095*	X:5261380-5279772	lncRNA
FBR2	1	0	LOC119069398**	X:15532893-15534365	upstream activation factor subunit spp27
FBR2	1	0	LOC119069400	X:15528862-15529735	uncharacterized LOC119069400
FBR2	1	0	LOC119069401	X:15521731-15523213	uncharacterized LOC119069401
FBR2	2	-99	LOC119069398**	X:15532893-15534365	upstream activation factor subunit spp27
FBR2	3	0	LOC119070108	X:15626340-15628004	uncharacterized LOC119070108
FBR3	1	0	LOC119085652	X:58156161-58159064	uncharacterized LOC119085652
FBR3	2	0	LOC119081069***	X:58188821-58243636	F-actin-monooxygenase Mical
FBR3	2	0	LOC119081098	X:58195622-58202717	carboxymethylenebutenolidase homolog
FBR3	3	0	LOC119081069***	X:58188821-58243636	F-actin-monooxygenase Mical
FBR3	3	0	LOC119081088	X:58245423-58256907	palmitoyltransferase ZDHHC6
3pINVbp	1	0	LOC119069629	X:62848697-62862727	rapamycin-insensitive companion of mTOR
3pINVbp	1	0	LOC119069633	X:62884193-62893941	histone-lysine N-methyltransferase Suv4-20
3pINVbp	1	0	LOC119069637	X:62865320-62877204	cytochrome P450 4d1-like
3pINVbp	1	0	LOC119069648	X:62864133-62865362	protein FAM32A-like

Interaction region is specific FBR (or long paracentric inversion breakpoint). Summit candidate is as defined for each interaction region in Supplemental Table S6. For each, summit candidate 1 had most support. The genes closest to each region are reported. Gene distance corresponds to how far away from the region coordinates the gene is, where 0 means it is within the region, negative numbers are upstream, and positive downstream of region boundaries. Gene ID corresponds to NCBI's RefSeq annotation of Bcop_v1, but the gene coordinates are shown for Bcop_v2. The associated functional information is simply what putative orthologous protein assignment was given by NBCI during their annotation process.

Supplemental Table S6. Approximate Mb Distance between interacting loci of "extra dots" on X and IV.

Dot	Approximate Mb Distance between interacting loci
X-dot-1	55
X-dot-2	41
X-dot-3	49
X-dot-4	39
X-dot-5	28
X-dot-6	29
X-dot-7	8
IV-dot-1	12

Interaction region is the X-dot name as given in Supplemental Fig S7. There is only one IV-dot.

Supplemental Table S7. Genes found within X-dot-1 interaction regions.

Interaction Region	Region Coordinates	Gene Distance	Gene ID	Gene Coordinates	Associated functional info.
X-dot-1-1	X:5720579-5789502	0	LOC119070124	X:5731320-5738467	protein UBASH3A homolog
X-dot-1-1	X:5720579-5789502	0	LOC119070142	X:5726821-5729416	histone deacetylase complex subunit SAP30 homolog
X-dot-1-1	X:5720579-5789502	0	LOC119070135	X:5750829-5754225	nuclear transcription factor Y subunit gamma-like
X-dot-1-1	X:5720579-5789502	0	LOC119070126	X:5707253-5722892	plastin-1
X-dot-1-1	X:5720579-5789502	0	LOC119070144	X:5739860-5744992	uncharacterized LOC119070144
X-dot-1-2	X:61065460-61134383	0	LOC119069759	X:61131487-61142319	lncRNA
X-dot-1-2	X:61065460-61134383	0	LOC119069757	X:61051512-61076636	lncRNA
X-dot-1-2	X:61065460-61134383	0	LOC119069730	X:61088186-61093172	uncharacterized LOC119069730
X-dot-1-2	X:61065460-61134383	0	LOC119069729	X:61067304-61080301	kinesin-like protein Klp98A

Interaction region is the X-dot name as given in Supplemental Fig S7, and whether it is the first or second region in the interaction. The coordinates of each region are given. These regions were the sub-regions of highest interaction frequency between the two loci. The genes closest to each region are reported. Gene distance corresponds to how far away from the region coordinates the gene is, where 0 means it is within the region. Gene ID corresponds to NCBI's RefSeq annotation of Bcop_v1, but the gene coordinates are shown for Bcop_v2. The associated functional information is simply what putative orthologous protein assignment was given by NBCI during their annotation process.

Supplemental Table S8. Genes found within X-dot-2 interaction regions.

Interaction Region	Region Coordinates	Gene Distance	Gene ID	Gene Coordinates	Associated functional info.
X-dot-2-1	X:11441158-11510081	0	LOC119069117	X:11502854-11509659	F-box/LRR-repeat protein fbxl-1
X-dot-2-1	X:11441158-11510081	0	LOC119069118	X:11495784-11500294	WD repeat domain phosphoinositide-interacting protein 3
X-dot-2-1	X:11441158-11510081	0	LOC119069119	X:11488255-11489402	tRNA-uridine aminocarboxypropyltransferase 1
X-dot-2-1	X:11441158-11510081	0	LOC119069120	X:11486337-11487001	signal peptidase complex subunit 1
X-dot-2-1	X:11441158-11510081	0	LOC119069121	X:11478332-11485466	band 4.1-like protein 5
X-dot-2-1	X:11441158-11510081	0	LOC119069122	X:11472499-11474482	actin-87E
X-dot-2-1	X:11441158-11510081	0	LOC119069124	X:11440885-11441749	uncharacterized LOC119069124
X-dot-2-2	X:52381207-52450130	0	LOC119085308	X:52429113-52433177	uncharacterized LOC119085308
X-dot-2-2	X:52381207-52450130	0	LOC119085312	X:52445680-52451577	lncRNA
X-dot-2-2	X:52381207-52450130	0	LOC119085329	X:52447651-52449164	lncRNA
X-dot-2-2	X:52381207-52450130	0	LOC119085335	X:52439152-52440564	lncRNA
X-dot-2-2	X:52381207-52450130	0	LOC119085343	X:52397951-52426733	putative thiamine transporter SLC35F3
X-dot-2-2	X:52381207-52450130	0	LOC119085423	X:52418394-52423109	uncharacterized LOC119085423
X-dot-2-2	X:52381207-52450130	0	LOC119085433	X:52394031-52396232	protein C3orf33
X-dot-2-2	X:52381207-52450130	0	LOC119085442	X:52382796-52393385	CTP synthase-like
X-dot-2-2	X:52381207-52450130	0	LOC119085467	X:52387325-52388438	lncRNA

Interaction region is the X-dot name as given in Supplemental Fig S7, and whether it is the first or second region in the interaction. The coordinates of each region are given. These regions were the sub-regions of highest interaction frequency between the two loci. The genes closest to each region are reported. Gene distance corresponds to how far away from the region coordinates the gene is, where 0 means it is within the region. Gene ID corresponds to NCBI's RefSeq annotation of Bcop_v1, but the gene coordinates are shown for Bcop_v2. The associated functional information is simply what putative orthologous protein assignment was given by NBCI during their annotation process.

Supplemental Table S9. Genes found within X-dot-3 interaction regions.

Interaction Region	Region Coordinates	Gene Distance	Gene ID	Gene Coordinates	Associated functional info.
X-dot-3-1	X:11441158-11510081	0	LOC119069117	X:11502854-11509659	F-box/LRR-repeat protein fbxl-1
X-dot-3-1	X:11441158-11510081	0	LOC119069118	X:11495784-11500294	WD repeat domain phosphoinositide-interacting protein 3
X-dot-3-1	X:11441158-11510081	0	LOC119069119	X:11488255-11489402	tRNA-uridine aminocarboxypropyltransferase 1
X-dot-3-1	X:11441158-11510081	0	LOC119069120	X:11486337-11487001	signal peptidase complex subunit 1
X-dot-3-1	X:11441158-11510081	0	LOC119069121	X:11478332-11485466	band 4.1-like protein 5
X-dot-3-1	X:11441158-11510081	0	LOC119069122	X:11472499-11474482	actin-87E
X-dot-3-1	X:11441158-11510081	0	LOC119069124	X:11440885-11441749	uncharacterized LOC119069124
X-dot-3-2	X:60031621-60100543	0	LOC119069764	X:60072417-60107238	serine/threonine-protein kinase 32A
X-dot-3-2	X:60031621-60100543	0	LOC119069765	X:59993213-60057104	inactive dipeptidyl peptidase 10-like
X-dot-3-2	X:60031621-60100543	0	LOC119069766	X:60042619-60043531	bombyxin C-1-like
X-dot-3-2	X:60031621-60100543	0	LOC119069767	X:60040039-60059176	lncRNA

Interaction region is the X-dot name as given in Supplemental Fig S7, and whether it is the first or second region in the interaction. The coordinates of each region are given. These regions were the sub-regions of highest interaction frequency between the two loci. The genes closest to each region are reported. Gene distance corresponds to how far away from the region coordinates the gene is, where 0 means it is within the region. Gene ID corresponds to NCBI's RefSeq annotation of Bcop_v1, but the gene coordinates are shown for Bcop_v2. The associated functional information is simply what putative orthologous protein assignment was given by NBCI during their annotation process.

Supplemental Table S10. Genes found within X-dot-4 interaction regions.

Interaction Region	Region Coordinates	Gene Distance	Gene ID	Gene Coordinates	Associated functional info.
X-dot-4-1	X:13577760-13646683	0	LOC119069895	X:13631028-13719221	sex determination protein fruitless
X-dot-4-1	X:13577760-13646683	0	LOC119069899	X:13619455-13620308	C-type lectin 37Db-like
X-dot-4-1	X:13577760-13646683	0	LOC119069903	X:13579837-13596288	lncRNA
X-dot-4-2	X:52656898-52725821	0	LOC119085108	X:52655868-52722399	uncharacterized LOC119085108
X-dot-4-2	X:52656898-52725821	0	LOC119085137	X:52639114-52740107	uncharacterized LOC119085137

Interaction region is the X-dot name as given in Supplemental Fig S7, and whether it is the first or second region in the interaction. The coordinates of each region are given. These regions were the sub-regions of highest interaction frequency between the two loci. The genes closest to each region are reported. Gene distance corresponds to how far away from the region coordinates the gene is, where 0 means it is within the region. Gene ID corresponds to NCBI's RefSeq annotation of Bcop_v1, but the gene coordinates are shown for Bcop_v2. The associated functional information is simply what putative orthologous protein assignment was given by NBCI during their annotation process.

Supplemental Table S11. Genes found within X-dot-5 interaction regions.

Interaction Region	Region Coordinates	Gene Distance	Gene ID	Gene Coordinates	Associated functional info.
X-dot-5-1	X:26052758-26121681	0	LOC119068645	X:26084527-26125653	proton channel OtopLc
X-dot-5-1	X:26052758-26121681	0	LOC119068646	X:26055260-26067017	uncharacterized LOC119068646
X-dot-5-2	X:54104273-54173196	0	LOC119084022	X:54083799-54218142	aminopeptidase N
X-dot-5-2	X:54104273-54173196	0	LOC119084034	X:54118226-54126574	myosin-1-like
X-dot-5-2	X:54104273-54173196	0	LOC119084041	X:54135371-54139896	uncharacterized LOC119084041
X-dot-5-2	X:54104273-54173196	0	LOC119084060	X:54106794-54111456	uncharacterized LOC119084060
X-dot-5-2	X:54104273-54173196	0	LOC119084116	X:54173106-54177132	uncharacterized LOC119084116
X-dot-5-2	X:54104273-54173196	0	LOC119084127	X:54168537-54170103	uncharacterized LOC119084127
X-dot-5-2	X:54104273-54173196	0	LOC119085667	X:54169975-54171186	uncharacterized LOC119085667
X-dot-5-2	X:54104273-54173196	0	LOC119085668	X:54133316-54134916	uncharacterized LOC119085668

Interaction region is the X-dot name as given in Supplemental Fig S7, and whether it is the first or second region in the interaction. The coordinates of each region are given. These regions were the sub-regions of highest interaction frequency between the two loci. The genes closest to each region are reported. Gene distance corresponds to how far away from the region coordinates the gene is, where 0 means it is within the region. Gene ID corresponds to NCBI's RefSeq annotation of Bcop_v1, but the gene coordinates are shown for Bcop_v2. The associated functional information is simply what putative orthologous protein assignment was given by NBCI during their annotation process.

Supplemental Table S12. Genes found within X-dot-6 interaction regions.

Interaction Region	Region Coordinates	Gene Distance	Gene ID	Gene Coordinates	Associated functional info.
X-dot-6-1	X:27086598-27155521	0	LOC119068853	X:27114485-27176903	partitioning defective 3 homolog
X-dot-6-1	X:27086598-27155521	0	LOC119068861	X:27104379-27107357	coiled-coil domain-containing protein 186
X-dot-6-1	X:27086598-27155521	0	LOC119068870	X:27102759-27109686	allantoinase
X-dot-6-1	X:27086598-27155521	0	LOC119068880	X:27110979-27112289	uncharacterized LOC119068880
X-dot-6-1	X:27086598-27155521	0	LOC119068881	X:27085637-27096036	cyclic AMP response element-binding protein B
X-dot-6-1	X:27086598-27155521	0	LOC119068895	X:27099377-27101069	lncRNA
X-dot-6-2	X:56240875-56309798	0	LOC119082597	X:56294010-56309659	progestin and adipoQ receptor family member 4
X-dot-6-2	X:56240875-56309798	0	LOC119085662	X:56226095-56248115	probable serine/threonine-protein kinase DDB_G0282963

Interaction region is the X-dot name as given in Supplemental Fig S7, and whether it is the first or second region in the interaction. The coordinates of each region are given. These regions were the sub-regions of highest interaction frequency between the two loci. The genes closest to each region are reported. Gene distance corresponds to how far away from the region coordinates the gene is, where 0 means it is within the region. Gene ID corresponds to NCBI's RefSeq annotation of Bcop_v1, but the gene coordinates are shown for Bcop_v2. The associated functional information is simply what putative orthologous protein assignment was given by NBCI during their annotation process.

Supplemental Table S13. Genes found within X-dot-7 interaction regions.

Interaction Region	Region Coordinates	Gene Distance	Gene ID	Gene Coordinates	Associated functional info.
X-dot-7-1	X:52312285-52381207	0	LOC119085485	X:52356775-52358594	uncharacterized LOC119085485
X-dot-7-1	X:52312285-52381207	0	LOC119085529	X:52273536-52316850	uncharacterized LOC119085529
X-dot-7-1	X:52312285-52381207	0	LOC119085513	X:52319529-52321223	uncharacterized LOC119085513
X-dot-7-1	X:52312285-52381207	0	LOC119085503	X:52325927-52328518	neprilysin-2-like
X-dot-7-1	X:52312285-52381207	0	LOC119085457	X:52378068-52379883	lncRNA
X-dot-7-1	X:52312285-52381207	0	LOC119085477	X:52366806-52368404	HSPB1-associated protein 1
X-dot-7-1	X:52312285-52381207	0	LOC119085676	X:52371744-52375851	ionotropic receptor 40a
X-dot-7-1	X:52312285-52381207	0	LOC119085495	X:52352709-52353674	copper metallothionein 2-like
X-dot-7-2	X:60031621-60100543	0	LOC119069767	X:60040039-60059176	lncRNA
X-dot-7-2	X:60031621-60100543	0	LOC119069766	X:60042619-60043531	bombyxin C-1-like
X-dot-7-2	X:60031621-60100543	0	LOC119069765	X:59993213-60057104	inactive dipeptidyl peptidase 10-like
X-dot-7-2	X:60031621-60100543	0	LOC119069764	X:60072417-60107238	serine/threonine-protein kinase 32A

Interaction region is the X-dot name as given in Supplemental Fig S7, and whether it is the first or second region in the interaction. The coordinates of each region are given. These regions were the sub-regions of highest interaction frequency between the two loci. The genes closest to each region are reported. Gene distance corresponds to how far away from the region coordinates the gene is, where 0 means it is within the region. Gene ID corresponds to NCBI's RefSeq annotation of Bcop_v1, but the gene coordinates are shown for Bcop_v2. The associated functional information is simply what putative orthologous protein assignment was given by NBCI during their annotation process.

Supplemental Table S14. Genes found within IV-dot-1 interaction regions.

Interaction Region	Region Coordinates	Gene Distance	Gene ID	Gene Coordinates	Associated functional info.
IV-dot-1-1	IV:16504291-16551717	0	LOC119070788	IV:16494459-16583728	schwannomin-interacting protein 1 homolog
IV-dot-1-1	IV:16504291-16551717	0	LOC119070790	IV:16527154-16528867	probable proline iminopeptidase
IV-dot-1-1	IV:16504291-16551717	0	LOC119070791	IV:16535626-16537156	probable proline iminopeptidase
IV-dot-1-1	IV:16504291-16551717	0	LOC119070792	IV:16523895-16539013	probable proline iminopeptidase
IV-dot-1-2	IV:28408248-28455674	0	LOC119075328	IV:28408885-28411082	benzoate 4-monooxygenase-like
IV-dot-1-2	IV:28408248-28455674	0	LOC119075328	IV:28408885-28411082	benzoate 4-monooxygenase-like
IV-dot-1-2	IV:28408248-28455674	0	LOC119075409	IV:28443702-28473808	dynein heavy chain 7 axonemal
IV-dot-1-2	IV:28408248-28455674	0	LOC119075409	IV:28443702-28473808	dynein heavy chain 7 axonemal
IV-dot-1-2	IV:28408248-28455674	0	LOC119075410	IV:28447205-28465067	lncRNA
IV-dot-1-2	IV:28408248-28455674	0	LOC119075410	IV:28447205-28465067	lncRNA

Interaction region is the IV-dot name – only one was explored. The coordinates of each region are given. These regions were the sub-regions of highest interaction frequency between the two loci. The genes closest to each region are reported. Gene distance corresponds to how far away from the region coordinates the gene is, where 0 means it is within the region. Gene ID corresponds to NCBI's RefSeq annotation of Bcop_v1, but the gene coordinates are shown for Bcop_v2. The associated functional information is simply what putative orthologous protein assignment was given by NBCI during their annotation process.

Supplemental References

1. Urban JM, Foulk MS, Bliss JE, Coleman CM, Lu N, Mazloom R, et al. High contiguity de novo genome assembly and DNA modification analyses for the fungus fly, Sciara coprophila, using single-molecule sequencing. BMC Genomics 2021 221. 2021;22:1–23.

2. Lieberman-Aiden E, Van Berkum NL, Williams L, Imakaev M, Ragoczy T, Telling A, et al. Comprehensive mapping of long-range interactions reveals folding principles of the human genome. Science (80-). 2009;326:289–93.

3. Urban JM, Bateman JR, Garza KR, Borden J, Jain J, Brown A, et al. Bradysia (Sciara) coprophila larvae up-regulate DNA repair pathways and down-regulate developmental regulators in response to ionizing radiation. Genetics. 2023. https://doi.org/10.1093/GENETICS/IYAD208.

4. Feyereisen R, Urban JM, Nelson DR. Aliens in the CYPome of the black fungus gnat, Bradysia coprophila. Insect Biochem Mol Biol. 2023;159:103965.

5. Urban JM. Bradysia coprophila genome annotations Bcop_v1.0. USDA Ag Data Commons. 2021. https://doi.org/10.15482/USDA.ADC/1522618.

6. NCBI. Bradysia coprophila Annotation Release 100. NCBI Eukaryotic Genome Annotation Pipeline. https://www.ncbi.nlm.nih.gov/genome/annotation_euk/Bradysia_coprophila/100/.

7. da Cunha PR, Granadino B, Perondini AL, Sánchez L. Dosage compensation in sciarids is achieved by hypertranscription of the single X chromosome in males. Genetics. 1994;138:787–90.

8. Baird RB, Urban JM, Mongue AJ, Jaron KS, Hodson CN, Grewoldt M, et al. Recent Evolution of a Maternally Acting Sex-Determining Supergene in a Fly with Single-Sex Broods. Mol Biol Evol. 2023;40.

9. Layer RM, Chiang C, Quinlan AR, Hall IM. LUMPY: a probabilistic framework for structural variant discovery. Genome Biol. 2014;15:R84.

10. Pedersen BS, R. L, Quilan A. smoove: structural-variant calling and genotyping with existing tools. Available from: https://github.com/brentp/smoove. 2020.
